# The role of tumor-associated macrophages in tumor vascularization

**DOI:** 10.1186/2045-824X-5-20

**Published:** 2013-12-06

**Authors:** Chunqing Guo, Annicole Buranych, Devanand Sarkar, Paul B Fisher, Xiang-Yang Wang

**Affiliations:** 1 Department of Human & Molecular Genetics, Virginia Commonwealth University School of Medicine, PO BOX 980033, Richmond VA23298, USA; 2 VCU Institute of Molecular Medicine, Virginia Commonwealth University School of Medicine, Richmond VA23298, USA; 3 VCU Massey Cancer Center, Virginia Commonwealth University School of Medicine, Richmond VA23298, USA

**Keywords:** Angiogenesis, Tumor vascularization, Tumor-associated macrophages

## Abstract

Tumor vascularization is a highly complex process that involves the interaction between tumors and their surrounding stroma, as well as many distinct angiogenesis-regulating factors. Tumor associated macrophages (TAMs) represent one of the most abundant cell components in the tumor environment and key contributors to cancer-related inflammation. A large body of evidence supports the notion that TAMs play a critical role in promoting the formation of an abnormal tumor vascular network and subsequent tumor progression and invasion. Clinical and experimental evidence has shown that high levels of infiltrating TAMs are associated with poor patient prognosis and tumor resistance to therapies. In addition to stimulating angiogenesis during tumor growth, TAMs enhance tumor revascularization in response to cytotoxic therapy (e.g., radiotherapy), thereby causing cancer relapse. In this review, we highlight the emerging data related to the phenotype and polarization of TAMs in the tumor microenvironment, as well as the underlying mechanisms of macrophage function in the regulation of the angiogenic switch and tumor vascularization. Additionally, we discuss the potential of targeting pro-angiogenic TAMs, or reprograming TAMs toward a tumoricidal and angiostatic phenotype, to promote normalization of the tumor vasculature to enhance the outcome of cancer therapies.

## Introduction

It is well known that progressive tumors require vascular development for delivery of oxygen and nourishment into the tumor to facilitate their survival, growth and capacity to metastasize [1]. Tumor vascularization, or angiogenesis, represents one of the hallmarks of cancer and plays an essential role in tumor progression, invasion and metastasis [2,3]. Blood vessels dramatically increase in most tumors during the tumor transition to malignant states, a process termed as the “angiogenic switch” [4,5]. Tumor vascularization is influenced by many molecular and cellular events in the tumor microenvironment (TME), since transformed cells secrete pro-angiogenic molecules that recruit and activate not only endothelial cells (ECs), but also stromal cells such as macrophages. Unlike physiological or developmental angiogenesis, tumor vasculatures are known to be structurally and functionally abnormal, characterized by poor blood flow, leakiness and dilation [4,5].

Macrophages are of the myeloid cell lineage and constitute the first line of innate defense against invading pathogens by engulfing microbes or presenting antigens to T cells [6]. They also play crucial roles in tissue homeostasis, repair, and remodeling via production of various cytokines, chemokines, growth factors and proteolytic enzymes [6-8]. An enhanced number of inflammatory leukocytes are often found in mouse and human tumors compared with surrounding normal tissues [9,10], suggesting a potential link between these cells and tumor vascularization. More specifically, the most abundant cell population among the inflammatory cells in the solid tumor environment, tumor associated macrophages (TAMs), have garnered considerable interest in recent years as key initiators of chronic inflammation in the TME by producing growth factors and inflammatory cytokines [11]. Accumulating evidence suggests that TAMs act as a key effectors, provoking a pro-angiogenic outcome during the “angiogenic switch” [12,13], and play a prominent role in stimulating tumor angiogenesis and progression [12,14].

## Macrophage polarization in the tumor environment

The highly malleable macrophages mainly originate from blood monocytes infiltrating peripheral tissues and subsequently acquire distinct characteristics as a result of environmental cues [6]. TAMs are reportedly present in both perivascular and hypoxic regions of different mouse and human tumors [15-17]. The TME often directs macrophage polarization from the M1 (classically activated) state, which is associated with an anti-angiogenic and anti-tumorigenic response, to the M2 (alternatively activated) state, a phenotype that promotes angiogenesis and tissue remodeling as well as immunosuppression [5,18,19]. TAMs phenotypically resemble M2-like macrophages due to their ability to secrete pro-angiogenic factors promoting tumor vascularization and induce development of abnormal vessels [20,21]. Murine TAMs display signature molecules of M2-like or alternatively activated macrophages, such as arginase-I, scavenger and mannose receptors, vascular endothelial growth factor (VEGF), matrix metalloproteinases (MMPs), osteopontin and transforming growth factor-β (TGF-β) [22-24]. In contrast, TAMs often display variable phenotypes depending on the stage of tumor development. For example, while TAMs are biased toward the M2-like state in advanced tumors, in early stages or in regressing tumors, TAMs tend to resemble the M1-like phenotype, further supporting angiogenesis inhibition and anti-tumor immunity by these pleiotropic cells [5,25].

Molecular profiling demonstrates that TAM subpopulations express both canonical M1 and M2 markers, although at significantly different levels [16,17,26]. Distinct subpopulations with a variably skewed M2-like phenotype coexist in mouse and human tumors [16,17,27]. Thus, it is conceivable that the dynamic changes in TAM phenotypes within the TME regulate the tumor vascular network, including angiogenesis and abnormal vessel development. The predictive value of M2-macrophage-associated markers (e.g., CD163) demonstrated in clinical studies also supports the notion that TAM polarization is of disease relevance [28]. Similar results obtained in mouse and clinical studies demonstrate that high macrophage frequency in many human cancer types closely correlates with increased tumor angiogenesis, metastasis, and poor prognosis [28-31].

While the correlation between TAMs and cancer prognosis or angiogenesis has been well described in different forms of human cancer [32], our understanding of the direct correlation between TAMs with an M2-like phenotype or characteristics and vascularization in human cancer is relatively limited. A few studies documented that the levels of CD163- or heme oxygenase-1-expressing macrophages are associated with the numbers of vessels in human intrahepatic cholangiocarcinoma [33] or glioma [34].

It was recently shown that both the origin and phenotype of TAMs might differ in primary tumors and metastases [35]. Such complexity emphasizes the diversity of TAM programming that is directed by the surrounding *milieu* within individual tumors [5,19,36,37]. Their dynamic interaction with the TME constantly shapes TAM phenotype and functioning, favoring tumor vascularization, invasion and subsequent metastasis. Therefore, M1/M2 classification of macrophages provides a useful working scheme; however, it is an oversimplification of the complexity of the functional states of macrophage activation as well as the heterogeneity and plasticity of macrophage in the TME.

The polarization of TAMs to a pro-angiogenic phenotype is regulated by multiple factors in the TME. For example, signals derived from stromal and inflammatory cells, hypoxia, genetic or epigenetic changes of cancer cells [20], as well as several molecular signaling pathways, including NF-κB [23,38], Notch [39] and Wnt5a [40], are important regulators of polarization of TAMs. Furthermore, transcription factors, such as signal transducer and activator of transcription 6 (STAT6), peroxisome proliferator-activated receptor-gamma (PPAR-γ), and c-Myc, are also involved in alternative activation of TAMs [41,42]. A recent study reported that macrophage-derived migration inhibitory factor (MIF) is an important determinant of the alternative activation of TAMs in melanoma-bearing mice [43]. MIF deficiency or treatment with a MIF antagonist attenuates tumor-induced TAM polarization and reduces the expression of pro-angiogenic genes in TAMs [43].

## Regulation of tumor vascularization by TAMs

Mononuclear phagocytic lineage cells, such as TAMs, are recognized as major contributors in the angiogenic process [5,44]. The potential role of macrophages in regulating tumor angiogenesis was initially proposed in the early 1990s [45]. The positive correlation between microvessel density and the level of infiltrating TAMs in tumor vessel areas, as well as poor prognosis in cancer patients, further supports the pro-angiogenic functions of these cells during human cancer progression [19,31,46]. Regulation of tumor vascularization by TAMs has been extensively investigated in animal tumor models [47-49].

When a mouse strain that develops oncogene-induced mammary tumors (MMTV-PyMT, mammary tumor virus promoter-driven polyoma middle T oncogene) was crossbred with mice carrying a homozygously mutated colony stimulating factor-1 (CSF-1) gene, the resulting ablation of macrophages delayed the angiogenic switch and tumor progression, whereas restoration of macrophage infiltration rescued the vessel phenotype [48]. Conversely, overexpression of the CSF-1 transgene in the mammary epithelium was found to promote the recruitment of monocyte/macrophages, which correlated with accelerated tumor progression in MMTV-PyMT mice in comparison to the nontransgenic counterparts [47]. Indeed, macrophages have a direct effect on the angiogenic switch (i.e., transition from a quiescent to a growing vasculature) and formation of the vessel network, subsequently accelerating the tumors’ progression to malignancy [1,48].

Extensive studies have established the roles of TAMs in promoting tumor angiogenesis or vascularization through their immense production of pro-angiogenic growth factors and cytokines. Transcriptional profiling analysis of late-stage mammary tumors from MMTV-PyMT mice documented that TAMs are highly enriched in transcripts encoding angiogenic factors, such as well-characterized VEGF, in comparison to a similar cell population from the spleens of non-tumor-bearing mice [50]. In tumor hypoxic areas, TAMs represent a critical source of VEGF-A, which functions as a potent mitogen for ECs by binding to VEGFR1/2 in human breast tumors [51]. Genetic studies indicated that VEGF-A produced by TAMs encompasses one of the essential factors involved in regulating the onset of the angiogenic switch and progression of MMTV-PyMT mammary mouse tumors [48,52,53]. Stockmann et al. recently showed that targeted ablation of the *vegfa* gene in myeloid cells attenuated the formation of what is typically a high-density vessel network, thus blocking the angiogenic switch in solid tumors [54]. However, the loss of VEGFA in tumor-infiltrating myeloid cells (the majority of which are TAMs) failed to inhibit the progression of subcutaneous and autochthonous (MMTV-PyMT) tumors, although it increased the susceptibility of tumors to chemotherapeutic cytotoxicity [54]. A recent study reported that depletion of TAMs reduced total *vegf* mRNA levels but did not affect vascular density in MMTV-PyMT tumors [55]. These studies suggest that VEGF-derived from other cell types in the TME, such as cancer cells [56], also contributes to tumor angiogenesis and progression. In addition, TAMs have the ability to produce a number of other pro-angiogenic factors, including growth factors and inflammatory cytokines or mediators, e.g., basic fibroblast growth factor (bFGF), macrophage-inhibitory factor, platelet activating factor, prostaglandin E2, osteopontin, adrenomedullin, PlGF, PDGF, TGF-β, IL-1β, IL-8 and TNF-α [57-61].

Tumor and inflammatory cells of the TME are surrounded by an extracellular matrix (ECM). TAMs affect the composition of the ECM by producing various matrix-remodeling proteolytic enzymes, such as MMP-2, MMP-7, MMP-9, MMP-12 [19,62]. TAMs also serve as the primary source for cathepsin protease activity in pancreatic cancer and mammary tumors; removal of TAM-derived cathepsin B or cathepsin S in these tumors impairs tumor angiogenesis [63,64]. The MMPs can induce degradation of the sustaining basement membrane and remodeling of ECM [65], thus promoting the migration and proliferation of ECs. MMP-9 also mobilizes the latent forms of VEGF sequestered in the ECM and enhances their bioavailability in RIP1-Tag2 mice, a pancreatic islet carcinogenesis model [66]. Indeed, MMP-9 produced by tumor infiltrating myeloid cells, including TAMs, or bone marrow (BM) cells is crucial for tumor angiogenesis and progression [66,67]. A subsequent study demonstrated that targeting macrophages expressing MMP-9 suppresses angiogenesis development in estrogen-treated K14-HPV16 transgenic mice, a model of human cervical carcinogenesis [68]. Two recent studies using mouse models of mammary carcinoma and glioblastoma (GBM) also support the essential role of MMP-9 when associated with BM cells or macrophages in increasing VEGF bioavailability and initiating tumor vascularization [69,70]. Thymidine phosphorylase, a pro-angiogenic enzyme expressed in TAMs, has also been associated with tumor vascularization and poor prognosis in cancer patients [71-74].

## Molecular pathways regulating the pro-angiogenic TAMs

TAMs are mobilized from the BM and recruited to the TME to promote tumor vascularization by tumor-derived cytokines or chemokines. CSF-1, also known as macrophage-colony stimulating factor (M-CSF), is the main regulator of the proliferation, differentiation, survival, and chemotaxis of monocytes/macrophages in tumor-bearing mice [6,47,75]. Depletion or inhibition of CSF-1 suppresses the infiltration of TAMs, which is associated with a significantly impaired tumor progression [47,75]. Recent studies demonstrated that VEGF-A is a potent chemoattractant for macrophages and that it can directly orchestrate the infiltration of monocytes/macrophages into tumors by engaging VEGFR1 signaling [76,77]. Monocyte chemoattractant protein-1 or (C-C motif) ligand 2 (MCP-1/CCL2) is a chemokine involved in recruiting monocytes to inflamed tissues [78]. MCP-1/CCL2 expression in human tumors correlates with monocyte/macrophage infiltration, as well as advanced tumor stages and metastatic relapse in breast cancer patients [79,80]. MCP-1/CCL2 can also stimulate macrophages to secrete urokinase-type plasminogen activator (uPAR) and MMP-9, both of which have the ability to remodel the tumor ECM [66,81]. In prostate cancer, recruiting pro-angiogenic macrophages into primary and metastatic tumors is one of the mechanisms by which MCP-1/CCL2 promotes tumorigenesis and metastasis [82]. Moreover, MCP-1/CCL2 and IL-6 induce an amplification loop that promotes TME-induced macrophage polarization toward the M2-like phenotype via the inhibition of caspase-8 cleavage and enhanced autophagy [83]. It is also worth noting that TAMs themselves are a rich source of various inflammatory chemokines. Thus, chemokines abundantly produced by TAMs also amplify the recruitment of myeloid cells, further extending the aberrant vascularization within the TME [11,84].

The chemokine (C-X-C motif) ligand 12 (i.e., CXCL12), also known as stromal cell-derived factor-1 (SDF-1), is expressed by tumor cells, fibroblasts and ECs within the tumors. Similar to VEGF, CXCL12 is highly upregulated in hypoxic tumors and provides a strong chemotactic signal for cells expressing CXCR4 or CXCR7, such as myeloid-lineage cells and ECs [70,85-88]. Interestingly, CD163^+^ perivascular macrophages in human metastatic melanoma express high levels of CXCL12 and autocrine CXCL12 production modulates the differentiation of monocytes toward a distinct program with pro-angiogenic functions, indicated by upregulation of VEGF and the angiogenic chemokine, CCL1 [89].

Placental growth factor (PlGF), a member of the VEGF family, can bind VEGFR1 and neuropilins expressed on ECs, macrophages and tumor cells [90]. The pro-angiogenic activity of PlGF in tumors is partially mediated by its ability to recruit VEGFR1^+^ monocytes/macrophages into tumors [58]. Blocking stromal- or tumor-produced PlGF inhibits tumor vascularization and TAM accumulation [58,91]. Deficiency of stromal PlGF alters the pro-angiogenic phenotype of TAMs and causes reduced tumor blood vessels [92].

The ability of TAMs to produce angiogenic factors is regulated by several transcription factors and signaling pathways. Activation of signal transducer and activator of transcription 3 (STAT3) mediates the function of TAMs in angiogenesis by upregulating several pro-angiogenic factors, e.g., VEGF and bFGF [93]. Tumor cell-derived soluble factors and direct cell-cell contact with tumors cells induce strong STAT3 activation in macrophages [93,94]. STAT3-regulated factors produced by both tumor cells and tumor-associated myeloid cells or TAMs also induced constitutive activation of STAT3 in tumor ECs, underscoring a central role of STAT3 signaling in mediating multidirectional crosstalk among tumor cells, myeloid cells and ECs in the TME that contributes to tumor angiogenesis [95].

The transcription factor Ets2 serves as a target for CSF-1 signaling pathways that regulate macrophage functions during inflammation [96,97]. Conditional ablation of Ets2 in TAMs results in decreased angiogenesis and reduced growth of mouse mammary tumors, as well as the reduced frequency and size of lung metastases, suggesting that Ets2 serves as the driver for a transcriptional program that promotes angiogenesis of breast tumors [98]. The Ets2 mechanism of action in TAMs is suggested to involve direct repression of anti-angiogenesis genes (*Thbs1, Thbs2, Timp1, and Timp3*) [98]. The NF-κB [99], TSC2–mTOR [100] and FLT-1 [101] signaling pathways also play important regulatory roles in the pro-angiogenic functions of TAMs.

Hypoxia is a common feature of solid tumors and a major driver of angiogenesis [102]. Many TAMs accumulate in hypoxic and/or necrotic areas of tumors, probably due to the release of hypoxia-induced chemoattractants such as VEGF and endothelins [81]. Upregulation of hypoxia-inducible factor-1α (HIF-1α) in the highly hypoxic GBMs results in the elevation of both VEGF and CXCL12, promoting the influx of BM-derived myeloid cells such as MMP-9-producing TAMs in the TME [70]. The knockdown of prolyl hyroxylase 2 (Phd2), a molecular oxygen sensor and negative regulator of HIF-1α, in human colon cancer increases the number of CD11b^+^ tumor-associated myeloid cells and promotes angiogenesis [103]. These findings highlight the important role of tumor hypoxia for the recruitment of pro-angiogenic myeloid cells, including TAMs. Once TAMs are recruited to the hypoxic areas, TAMs respond to hypoxia by upregulating hypoxia-inducible transcription factors (e.g., HIF-1α) for metabolic adaption, leading to an increase in transcription of a number of genes (e.g., VEGF, CXCL8) involved in regulating tumor vascularization [51,70,104]. In addition, TAMs also promote angiogenesis in the hypoxic condition by suppressing the expression of angiogenesis inhibitors, e.g., vasohibin-2 [105].

Several findings support a causal relationship between STAT3 activation and HIF-1α-dependent angiogenesis. STAT3 has been shown to be an important regulator of HIF-1α expression under both hypoxia and growth signaling conditions [106-108]. Activated STAT3 increases HIF-1α protein levels by blocking degradation or enhancing its de novo synthesis, which in turn enhances VEGF expression [109]. A novel autocrine loop (IL-6/STAT3/HIF-1α) that operates in cancer cells was recently discovered [110,111]. Interestingly, elevated STAT3 activity can increase HIF-1α promoter activity in both cancer cells and nontransformed, tumor-associated myeloid cells in the TME [107].

## TAM-related myeloid cells in tumor vascularization

Studies in mice have shown that tumors can recruit large numbers of monocytes, commonly regarded as the prospective TAM precursors, by secreting chemokines [12,112]. Upon differentiation into TAMs, these cells promote tumor growth, invasion, and metastasis by supporting the proliferation, survival, and motility of transformed cells, as well as tumor vascularization and suppression of antitumor immunity [35,36]. Although it has been reported that monocytes proliferate within tumors to generate TAMs [16], it is still unclear whether Ly6C^+^ “inflammatory monocytes” or Ly6C^–^ “resident monocytes” [113] are the primary source of TAMs in mice [16,114]. Therefore, TAMs originate from myeloid progenitors in response to tumor-secreted soluble factors, although the origin of TAMs in human cancer remains unclear.

A subpopulation of myeloid cells characterized by their expression of the angiopoietin receptor Tie2, also known as Tie2 expressing monocytes/macrophages (TEMs), has been identified in both human and murine tumors [115-117]. TEMs preferentially localize in the vicinity of tumor blood vessels [115-117]. Co-injection of tumor cells and TEMs derived from mouse mammary tumors into mice enhances tumor vascularization compared to their Tie2^−^ counterparts, while elimination of these cells using a suicide gene strategy significantly impairs tumor angiogenesis in subcutaneous mammary tumors or orthotopic human gliomas [115]. Similarly, human TEMs also provoke marked vascularization of human gliomas grown subcutaneously in nude mice [116], suggesting a fundamental role of TEMs in regulating angiogenesis. In addition, it has recently been suggested to use TEM frequency as a diagnostic marker for angiogenesis in hepatocellular carcinoma, potentially reflecting angiogenesis in the liver [118]. Gene expression profiling analyses show that tumor-derived TEMs are a subset of TAMs expressing a distinct gene signature consistent with enhanced pro-angiogenic/tissue-remodeling activity and lower pro-inflammatory activity [17]. Nonetheless, TEMs display an M2-like macrophage polarization, indicated by the enhanced expression of several scavenger receptors, including hemoglobin/haptoglobin scavenger receptor (*Cd163*), scavenger receptor A (*SRA* or *CD204*), mannose receptor (*MRC1* or *CD206*), hyaluronan receptor-1 (*Lyve1*), the lower expression of pro-inflammatory factors, e.g., interleukin 1β (*Il1b*) and nitric oxide synthase-2 (*Nos2*), and anti-angiogenic mediators, e.g., interleukin 12 (*Il12*) and *Cxcl10*[17,37].

Angiopoietins (ANGs) interactions with their receptor Tie2 are shown to be an emerging regulator of leukocyte trafficking and function in tumors [119]. Overexpression of ANG2 in the tumor vasculature induces the direct chemo-attraction of TEMs, indicated by enhanced recruitment of TEMs and consequently increased microvessel density in tumors [120]. ANG-2 markedly enhanced the pro-angiogenic activity of TEMs and increased their expression of two pro-angiogenic enzymes: thymidine phosphorylase and cathepsin B [105]. Additional studies using the approaches of ANG2 blockade or Tie2 knock-down in MMTV-PyMT mammary carcinomas and RIP1-Tag2 pancreatic insulinomas suggest that the surface levels of Tie2 in TEMs or ANG2-Tie2 signaling is required for TEM interactions with adjacent tumor blood vessels and subsequent tumor vascularization [15].

Tumors also recruit and expand myeloid-derived suppressor cells (MDSCs), a heterogeneous population of immature myeloid cells that are commonly identified by their expression of Gr-1 (Ly6C/G) and immunosuppressive activity [121,122]. Co-injection of MDSCs from murine tumors significantly increases the growth rate and blood vessel density of subcutaneous MC26 colorectal tumors [123]. Both MDSCs and TAMs have a phenotype similar to that of alternatively activated macrophages in the mouse [124]. STAT3 is suggested to contribute to the pro-angiogenic phenotype of TAMs and MDSCs [93]. Several lines of evidence suggest that MDSCs can mature into TAMs [125,126]. Interestingly, the crosstalk between MDSCs and TAMs results in increased production of MDSC-derived IL-10 and decreased production of IL-12 by TAMs, which further promotes tumor progression [127]. A recent study showed that hypoxia alters the function of MDSCs in the TME via HIF-1α and redirects their differentiation toward TAMs [128]. In addition to tumor cells, vascular ECs in the perivascular microenvironment can produce CSF1 and promote the functional polarization of M2-like macrophages that accelerate angiogenesis and tumor growth [129].

## TAMs, tumor vasculature and therapeutic response

Although tumor angiogenesis provides a promising target for the potential treatment of cancer, studies in mice and cancer patients have shown that anti-angiogenic therapies interfering with the VEGF pathway rarely induce long-lasting tumor responses [130], possibly due to the activation of VEGF-independent tumor vascularization [131]. Tumor hypoxia induced by anti-angiogenic treatment may promote the recruitment of BM-derived myeloid cells, including TEMs, to the tumors through chemotactic factors [131-133]. Therefore, the enhanced mobilization of myeloid cells, or TAMs, and their subsequent recruitment to the tumors are likely to contribute to the compensatory or alternative pro-angiogenic programs that render a tumor refractory to the anti-angiogenic blockade by VEGF antibodies [133]. Sorafenib, a small molecule inhibitor of tyrosine protein kinases, e.g., VEGF receptor 2 (VEGFR2), platelet derived growth factor receptor (PDGFR), and Raf kinases, also promotes TAM infiltration and elevation of CSF-1, SDF-1α/CXCL12 and VEGF in the tumors of hepatocellular carcinoma xenografts [49]. Elimination of TAMs with clodrolip (clodronate-containing liposomes) or Zoledronic acid strongly enhances sorafenib inhibited tumor progression and angiogenesis compared to mice treated with sorafenib alone [49]. Additionally, TAM depletion or CSF1R inhibitor synergizes with the anti-angiogenic effects of VEGF/VEGFR2 antibodies in controlling subcutaneous human cancer xenografts [134,135].

The ability of myeloid cells, including TAMs, to noticeably limit the efficacy of anti-angiogenic therapies was recently observed in mice treated with vascular-disrupting agents (VDAs) that selectively cause the transient collapse of tumor vasculature in order to achieve tumor destruction. However, concomitant tumor hypoxia and necrosis are accompanied with increased CXCL12 production and TEM infiltration in mouse mammary tumor models [86]. Blocking the recruitment of TEM using a CXCR4 antagonist or genetic ablation of TEM in tumor-bearing mice significantly enhances the efficacy of a VDA, i.e., combretastatin A4 phosphate [86].

TAMs and related myeloid cells are also associated with the failure of other cancer therapies. Several lines of evidence show that certain chemotherapeutic drugs enhance tumor recruitment of myeloid cells, e.g., TAMs, therefore limiting therapeutic outcomes. In a chemoresistant MCF-7 breast cancer model, combined chemotherapy (cyclophosphamide, methotrexate, and 5-fluorouracil), when used in conjunction with anti-CSF-1 antibodies, displayed markedly enhanced antitumor efficacy [136]. The CSF-1 blockade reduced TAM recruitment and angiogenesis, as well as down-regulated MMP-2 and MMP-12 expression in the tumor [136]. In the MMTV-PyMT mammary tumor model, inhibiting TAM recruitment using a selective CSF-1R inhibitor decreased blood vessel density and enhanced the efficacy of paclitaxel, a first-line treatment for metastatic breast cancer [55]. Additionally, this study underscores the prognostic value of the inverse correlation between the number of TAMs and cytotoxic T cells in breast cancer patients [55]. Therefore, a high TAM concentration promotes the formation of aberrant, hypo-perfused tumor vasculature that limits the delivery of chemotherapeutic agents into tumors. Furthermore, the ability of tumor-infiltrating TAMs to promote tumor chemoresistance is, at least in part, due to their suppression of the cytotoxic functions of effector T cells. It was recently shown that Trabectedin, a DNA-damaging agent approved for soft tissue sarcomas, inhibited the growth of mouse fibrosarcomas mainly by depleting monocytes and TAMs [137], suggesting that the antitumor efficacy of certain cytotoxic agents may partially rely on their ability to deplete pro-tumoral myeloid cells.

Radiotherapy (RT) is commonly used for treatment of many human cancers. In addition to the tumor ECs [138], emerging data underscores a possible role of tumor-infiltrating leukocytes in the regulation of tumor responses to RT [139]. Previous studies indicate a correlation between high TAM numbers and poor tumor responses to irradiation in mouse tumors [140]. In a mouse model of orthotopic human GBM, local RT and consequent vascular destruction promotes the recruitment of CD11b^+^ monocytes/macrophages via the up-regulation of HIF1α [87]. These myeloid cells mainly expressed F4/80 and Tie2 and were shown to promote tumor revascularization and relapse [87]. Blocking CXCL12 inhibited the recruitment of these myeloid-cells in response to RT and subsequently promoted the recovery of tumor vasculature, as well as the regrowth of irradiated tumors [87]. The same monocyte/macrophage cell population was also shown to promote tumor recurrence post-RT in a model of human head and neck squamous carcinoma in immune deficient mice [141]. It was also found that the use of anti-CD11b antibodies dramatically reduced myeloid cell infiltration and enhanced tumor responses to RT [141]. A subsequent study indicated that TEMs represent a major proportion of the myeloid cells recruited and localized around the tumor blood vessels after tumor irradiation [88]. It is proposed that these cells play a key role in facilitating tumor recurrence by promoting the survival of ECs and subsequent tumor revascularization. Targeting TAM or TAM-associated signaling to enhance the potency of RT has been similarly demonstrated in several other studies [142,143]. In addition to the rapid recruitment of TAMs, the irradiated TME also favors the polarization of M2-like macrophages that locate in avascular, hypoxic areas [142]. Thus, the recruited TAMs in irradiated TME are functionally similar to those of M2-like macrophages driving tissue repair during wound healing.

## Reprogramming TAMs to normalize tumor vasculature for improved anticancer therapy

There is an increasing amount of evidence supporting the concept of targeting TAMs or blocking the pro-angiogenic activity of TAMs to inhibit tumor vascularization and improve the therapeutic index of conventional cancer therapies [37,144]. Given the fact that macrophages of certain phenotypes possess the intrinsic ability to destroy cancer cells [5], reprograming pro-tumoral TAMs toward an anti-tumoral phenotype may represent a strategy to inhibit angiogenesis and provoke anti-tumor responses.

TNF-α is highly expressed by many human tumor types and plays a critical role in the induction of the pro-angiogenic phenotype of macrophages [145,146]. Eliminating leukocyte-derived TNF-α results in diffused vascular hemorrhage, stromal necrosis, and reduced tumor growth in MMTV-NeuT mice [147]. In addition, blocking TNF-α skews tumor-associated MRC1^+^Tie2^+^ TAMs from a pro-angiogenic phenotype to a pro-inflammatory/angiostatic phenotype, indicated by the upregulation of IL-12. Specific inhibition of the transcription factor NF-κB signaling in TAMs stimulates them to convert into classically activated cytotoxic cells, characterized by elevated IL-12 and MHC II expression [38]. The regression of tumors caused by TAM phenotypic changes depends on the tumoricidal activity of macrophages and natural killer cells [38]. In addition, tumor-targeted delivery of Th1 cytokine IFN-α using TEMs has been shown to reprogram TAMs toward a pro-inflammatory phenotype, inducing vascular normalization and impairing the growth of orthotopic gliomas and MMTV-PyMT mammary carcinomas [148]. Reprograming of TAMs with IFN-α also leads to a significant increase in CD11c^+^ macrophages or dendritic cells and provokes antitumor immune responses [148].

B lymphocytes and secreted immunoglobulins G (IgGs) were recently shown to promote skin carcinogenesis in K14-HPV16 mice through their interactions with immunoglobulin receptors (FcγR) expressed on tumor-infiltrating myeloid cells [149]. The absence of FcγR shifts TAMs from a pro-tumoral to an anti-tumoral phenotype, as indicated by an upregulation of “M1-like phenotype” genes (e.g., *Il1b*, *Il1a*, *Nos2*, *Il12a*, *Cxcl10*, *Cxcl11*) and a downregulation of genes associated with macrophages with “M2-like phenotype” or alternative activation (e.g., *Cd163*, *Il13*, *Il4*, *Ccl17*). In mice that are prone to skin-tumors, the lack of FcγR results in a reduced angiogenic response, as well as a reduced incidence of squamous cell carcinoma [149].

In addition to facilitating tumor angiogenesis, TAMs also induce abnormal tumor vessels in the hypoxic TME [32], thereby rendering tumors more resistant to cytotoxic therapies [150-152]. Thus, TAM-targeted therapy, such as TAM polarization, may potentially result in anti-angiogenic vessel normalization that not only reduces the aggressive phenotype of tumors, but also substantially enhances the therapeutic potency of other cancer treatments [151,152]. TAM depletion increased chemotherapeutic efficacy has been, at least partially, attributed to the normalization of blood vessels and improved delivery of therapeutic drugs [55,144].

Histidine-rich glycoprotein (HRG) is a heparin-binding plasma protein with anti-angiogenic activities, and its expression is downregulated in tumors. Intriguingly, HRG is highly effective in inducing M1-like polarization of TAMs by downregulating PIGF, as indicated by an increased production of angiostatic cytokines (e.g., IFN-β, CXCL10 and IL-12) and a concomitantly decreased expression of pro-angiogenic cytokines (e.g., CCL22, IL-1β and TNF-α) [92]. Skewing TAMs toward a pro-inflammatory phenotype by HRG treatment resulted in reduced vascular hypertrophy, dilation, tortuosity, and leakiness in multiple tumor models, therefore indicating a possible link between TAM polarization and vessel normalization. In addition, TAM polarization strongly augments antitumor immune responses and improves the antitumor efficacy of suboptimal doses of chemotherapeutic drugs, i.e., doxorubicin [92]. This study provides the first experimental evidence linking TAM polarization with normalization of tumor vasculature, highlighting the concept of reprograming TAMs as a novel strategy to improve other cancer therapies, such as chemotherapy and immunotherapy.

## Conclusions

**Figure 1:**
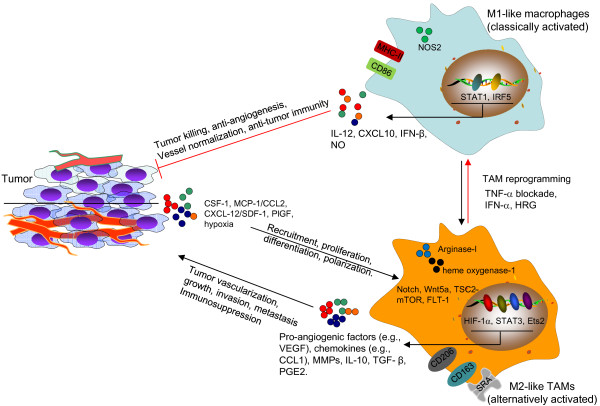
**Targeting TAMs to disrupt or normalize tumor vasculature.** Tumor cell-derived factors (MCP-1, SDF-1), multiple signaling pathways (Notch, Wnt5a, TSC2-mTOR and FLT-1) and transcription factors (HIF-1α, STAT3, Ets2) in the tumor environment recruit and/or polarize TAMs to an M2 (alternatively activated) state. TAMs produce pro-angiogenic factors and MMPs to promote the tumor vascularization during tumor growth and progression. TAMs and aberrant tumor vasculature also contribute to the failure of anticancer treatments, such as anti-angiogenesis therapy, chemotherapy and radiation therapy. TAM-targeted therapies can be designed to block the recruitment or pro-angiogenic activity of TAMs. TAMs can also be “re-educated” and reprogrammed to become antitumor effector cells with an M1-like phenotype, characterized by high expression of CD86, MHC-II and NOS2, enhanced production of IL-12, CXCL10, IFN-β and NO. These classically activated macrophages display anti-angiogenic, tumoricidal and immunostimulatory activities, facilitating the eradication of cancer cells. Targeting of TAMs may also potentially lead to the normalization of tumor vasculature, which synergizes with antitumor efficacy of other cytotoxic treatments, such as chemotherapy. HIF-1α, hypoxia-inducible factor-1α; HRG, Histidine-rich glycoprotein; IRF5, interferon regulatory factor 5; MCP-1, monocyte chemoattractant protein 1; MMP, matrix metalloproteinase; NO, nitric oxide; NOS2, nitric oxide synthase 2; PGE2, prostaglandin E2; SDF-1, stromal cell-derived factor-1; SRA, scavenger receptor A; STAT, Signal transducer and activator of transcription; TGF-β, transforming growth factor-β; VEGF, vascular endothelial growth factor.

Although hypoxia and VEGF are well-recognized as tumor-derived or intrinsic signals in promoting tumor vascularization, the crucial roles of non-maliganant cells within the TME in orchestrating this complex process has only recently been appreciated. A growing body of evidence indicates that TAMs, heterogeneous and functionally distinct myeloid cells, are directly involved in the tumor “angiogenic switch” and excessive tumor vascularization. Mobilization of macrophages and their polarization toward an alternatively activated or M2-like phenotype not only contributes to tumor growth, progression and invasion, but also negatively influences tumor responses to anti-angiogenic or anti-vascular treatments, and cytotoxic therapies. Myeloid cells, especially TAMs, promote abnormal blood vessel formation that subsequently limit chemotherapeutic efficacy [144]. Additionally, TAMs function as important participants in tumor revascularization following RT and facilitate cancer relapse [139]. Therefore, targeting TAMs by blocking their pro-angiogenic functions or reprogramming them toward an angiostatic, tumoricidal and immunostimulatory phenotype represent a potentially novel strategy in anti-angiogenic therapies and other conventional cancer treatments (Figure 1). Considering TAM polarization in the TME, “re-educating” and reprogramming TAMs to convert them into antitumor effectors is now emerging as a novel approach for “normalizing” tumor vasculature and remodeling the immune microenvironment. See a recent review by Squadrito and De Palma on pro-angiogenic macrophage and cancer therapy [153] for more details. These TAM-targeted strategies are being tested in preclinical and clinical settings for their use in conjunction with conventional cancer treatment modalities, such as chemotherapy, RT or immunotherapy, to achieve improved therapeutic efficacy. In addition, immune-based approaches to redirect the TAM phenotype for tumor rejection are clearly worth pursuing [154]. Nonetheless, much work remains in order to define and elucidate the mechanistic basis of TAM polarization and vessel normalization in the TME, which may lead to the identification of novel targets for therapeutic intervention of tumor vascularization or “re-education” of TAMs. Continuing research to understand the interactions between cancer cells and stromal cells, including TAMs or other myeloid cells, in the TME are fundamental to the rational design of future cancer treatments.

## Abbreviations

ANGs: Angiopoietins; BM: Bone marrow; CSF1: Colony stimulating factor-1; EC: Endothelial cell; ECM: Extracellular matrix; HRG: Histidine-rich glycoprotein; IFN-α: Interferon-α; IGF1: Insulin growth factor 1; IgGs: Immunoglobulins G; FGF: Fibroblast growth factor; HIF-1α: Hypoxia-inducible factor 1-α; M-CSF: Macrophage-colony stimulating factor; MCP-1: Monocyte chemoattractant protein 1; MDSC: Myeloid-derived suppressor cell; MIF: Migration inhibitory factor; MRC1: Mannose receptor, C type 1; MMP: Matrix metalloproteinase; MMTV-PyMT: Mammary tumor virus promoter-driven polyoma middle T oncogene; uPAR: Urokinase-type plasminogen activator; PDGFR: Platelet derived growth factor receptor; PlGF: Placental growth factor; RT: Radiotherapy; SDF-1: Stromal cell-derived factor-1; SRA: Scavenger receptor A; STAT: Signal transducer and activator of transcription; TAM: Tumor associated macrophage; TEM: Tie2 expressing monocyte/macrophage; TME: Tumor microenvironment; TNF-α: Tumor necrosis factor-α; VEGF: Vascular endothelial growth factor.

## Competing interests

The authors declare that they have no competing interests.

## Authors’ contributions

CG, AB, DS, PF and XW prepared and wrote the manuscript. All authors read and approved the final manuscript.
