# Correction: The role of tumor-associated macrophages in tumor vascularization

**DOI:** 10.1186/2045-824X-6-2

**Published:** 2014-02-10

**Authors:** Chunqing Guo, Annicole Buranych, Devanand Sarkar, Paul B Fisher, Xiang-Yang Wang

**Affiliations:** 1 Department of Human & Molecular Genetics, Virginia Commonwealth University School of Medicine, PO BOX 980033, Richmond VA23298, USA; 2 VCU Institute of Molecular Medicine, Virginia Commonwealth University School of Medicine, Richmond VA23298, USA; 3 VCU Massey Cancer Center, Virginia Commonwealth University School of Medicine, Richmond VA23298, USA

## Correction

Following publication of this article [1] it came to our attention that we neglected to acknowledge the inspiration for our review provided by earlier critical reviews in this area by Michele De Palma and colleagues [2,3]. We sincerely apologize for this oversight.
